# Baricitinib retention and factors associated thereof in clinical practice: a prospective, multicenter 12-month study in rheumatoid arthritis patients

**DOI:** 10.1007/s00296-026-06313-y

**Published:** 2026-10-05

**Authors:** Irini Flouri, Antonios Bertsias, Konstantina Zoupidou, Christos Koutsianas, George Fragoulis, Dimitrios Nezis-Katsifis, Elena Sambatakaki, Nestor Avgoustidis, Argyro Repa, George Bertsias, Maria Tektonidou, Petros Sfikakis, Dimitrios Vassilopoulos, Dimitrios Boumpas, Prodromos Sidiropoulos

**Affiliations:** 1 Rheumatology, Clinical Immunology, Medical School, University of Crete, Heraklion, Greece; 2 Joint Rheumatology Program, 4th Department of Medicine, School of Medicine, National and Kapodistrian University of Athens, Attikon General Hospital, Athens, Greece; 3 Joint Rheumatology Program, Clinical Immunology-Rheumatology Unit, 2nd Department of Medicine and Laboratory, School of Medicine, National and Kapodistrian University of Athens, General Hospital of Athens ‘Hippokration’, Athens, Greece; 4 Joint Academic Rheumatology Program, 1st Department of Propedeutic and Internal Medicine, National and Kapodistrian University of Greece, Athens, Greece; 5 Institute of Molecular Biology and Biotechnology, Foundation for Research and Technology Hellas (FORTH), Heraklion, Greece

**Keywords:** Arthritis, Rheumatoid, Janus kinase inhibitors

## Abstract

Herein we report for baricitinib drug retention, predictors thereof and clinical responses in rheumatoid arthritis (RA) patients with a special focus in comorbidities. Prospective, multicenter, observational study of RA patients starting baricitinib because of active disease. Patients were followed every 3 months for 12 months. The primary endpoint was baricitinib retention at 12 months; predictors for survival and clinical responses at 6 months were secondary endpoints. Retention rate was estimated by Kaplan–Meier (K-M) while predictors of discontinuation by Cox regression. We recruited 135 patients, 93.3% females, mean (standard deviation-SD) age 56.1 (10.1) years, median (interquartile range) disease duration 57 (90) months. 18.5% started baricitinib as first line targeted therapy. 35.9% (28/88) and 44.1% (26/59) achieved DAS28 remission/low disease activity (LDA) at 6 and 12 months respectively. Baricitinib retention at 12 months was 68.9%. K-M analysis showed that hypertension (p=0.049), latent tuberculosis infection (LTBi) (p=0.007) and depression (p=0.005) were associated to lower retention, while multivariate analysis showed that depression [Hazard ratio (HR) 2.715, p=0.007] and LTBi (HR 2.519, p=0.020) predicted baricitinib discontinuation. Analysis for patients on therapy for at least 6 months showed that together with hypertension (HR 2.477, p=0.022) and LTBi (HR 3.761, p=0.023), higher DAS28 at 6 months (HR 1.434, p=0.024) predicted baricitinib discontinuation at 12 months. In established RA, 68.9% of the patients remained on baricitinib at 12 months and had an improvement of disease activity. Mostly comorbidities contributed to baricitinib persistence, supporting their importance as factors to be addressed to optimize RA clinical care.

**Supplementary Information:**

The online version contains supplementary material available at https://doi.org/10.1007/s00296-026-06313-y.

## Introduction

Treatment options for rheumatoid arthritis (RA) have been expanded with the addition to the biologic disease modifying drugs (bDMARDs) those of targeted synthetic DMARDs (tsDMARDs) [1]. Baricitinib is a Janus kinase inhibitor (JAKi), proven effective as monotherapy or combined with conventional synthetic DMARDs (csDMARDs) across all treatment lines in randomized clinical trials (RCTs) [2–4]. Safety concerns for cardiovascular disease (CVD) and venous thromboembolism (VTE) identified in the “ORAL Surveillance” trial for tofacitinib, have prompted regulatory bodies to recommend careful, individualized risk assessment before starting JAKi therapy, particularly in patients with cardiovascular risk factors [1, 5]. Nevertheless, these initially raised concerns have not been further confirmed by large observational and registry-based studies, underscoring the value of real-life data for the better clinical evaluation of novel therapies [6–9].

Long-term extension data of baricitinib RCTs confirmed sustained response and radiographic damage protection over several years [2, 10–12]. Nevertheless, these data are based on patients selected for RCTs who differ from routine-care patients. Baricitinib effectiveness and retention in clinical practice have been described by analysis from international registries (eg, RA-BE-REAL, BSRBR-RA, ORBIT-RA) [13–15] still, there is not much information about how disease related characteristics and comorbidities influence drug survival.

This study aimed to describe baricitinib drug survival and predictors thereof as well as clinical effectiveness/safety in a real-world cohort of patients with RA followed over 12 months, and to identify demographic, disease-related and comorbid predictors of treatment discontinuation.

## Methods

### Patients and follow-up

This was a prospective, observational study of four collaborative academic rheumatology centers in Greece [University Hospital of Heraklion (Crete), “Attikon” Hospital (Athens), “Ippokratio” Hospital (Athens), “Laiko” hospital (Athens)]. Recruitment period started on March 2022 and lasted for 12 months, while follow-up of the patients on treatment was 12 months or till the date of discontinuation whichever came earlier. All patients signed informed consent form and the study was approved by local review boards of each participating center.

Patients recruited had a diagnosis of RA (according to the 2010 ACR/EULAR criteria) and initiated baricitinib (4 mg/day) due to active disease and according to national and EULAR guidelines for the treatment of RA. There were no limitations on background therapies or concomitant to baricitinib therapies for RA.

Demographic, disease related characteristics and comorbidities were recorded at baseline. Patients were followed every 3 months for the 12 months of treatment. Disease related characteristics [disease activity score of 28 joints (DAS28), 28 swollen joint count (28SJC), 28 tender joint count (28TJC), visual analog score (VAS) global, VAS pain, VAS physician, erythrocyte sedimentation rate (ESR) and C-reactive protein (CRP)], and function [modified Health Assessment Questionnaire (mHAQ)] were recorded in every follow-up visit. Disease activity categories were divided according to DAS28-ESR in high (HDA, DAS28>5.1), moderate (MDA, 3.2<DAS28 *≤ * 5.1), low disease activity (LDA, 2.6<DAS28 *≤ * 3.2) and remission (REM, DAS28 *≤ * 2.6).

Detailed data regarding rheumatologic drugs and their dosages, as well as treatment discontinuations and all adverse events (based on MedDRA coding) during follow-up, were also recorded. All data anonymized were recorded in a web-based registry platform (University of Crete Rheumatology Clinic Registry-UCRC) [16].

### Statistical analysis

All variables were summarized using descriptive statistics. For categorical variables the counts and respective percentages are presented. Continuous variables were summarized using mean (standard deviation) in cases of normally distributed data and median (min, max, IQR) in cases of non-normally distributed data. Univariate comparisons were performed using Pearson’s Chi-square test, Independent Samples T—Test (in case of normally distributed data) or the non-parametric Mann–Whitney Test in cases of non-normally distributed data. Differences in disease activity-related measurements (i.e. DAS28 ESR, SDAI, CDAI, mHAQ, VAS general patient, ESR and CRP) at 6 and 12 months of observation compared to baseline, were performed using linear mixed with dependent variables the aforementioned measurements, treating patient-level as random effect and time as fixed effect. Mean differences and the respective 95% confidence intervals are reported using an intention-to-treat analysis. Univariate comparisons in drug survival time and selected factors were performed using Kaplan–Meier test. Predictors of 12 month drug survival were assessed using Cox proportional hazards regression with backward stepwise selection (conditional likelihood ratio method, removal criterion p > 0.10). The initial model included as independent variables those identified as significant at univariate level. To this end, included variables besides gender (male vs female) and age at baricitinib start were the line of treatment (1–3 vs ≥4), baricitinib as monotherapy (yes/no), DAS28 ESR score at 6 months of drug intake, presence of hypertension (yes/no), presence of depression (yes/no) and presence of latent tuberculosis infection (LTBi) (yes/no). In order to account for disease activity levels at 6 months, the above analyses (both univariate and multivariate) were repeated selecting the subgroup of patients remained on baricitinib for >6 months. In all the above survival models, discontinuation was coded as an event. Time-to-event was calculated in months from the date of treatment initiation to the date of discontinuation (for patients who discontinued) or to the date of last follow-up or 12 months, whichever occurred first (for censored patients). For backward Cox regressions, model selection was guided by maintaining an events-per-variable ratio of at least 10, and model stability was further checked using influence diagnostics (DFBeta) to confirm that no individual patient was disproportionately affecting the results. Incidence rates of adverse events were calculated as the number of events divided by the total person-years of follow-up. The 95% confidence intervals were estimated assuming a Poisson distribution for the observed event counts. The level of significance was set to α=0.05 and the statistical analysis were performed using the statistical software IBM SPSS v. 26.

## Results

### Baseline patients characteristics

We recruited 135 RA patients, mostly females (93.3%), of mean (Standard Deviation-SD) age 56.1 (10.1) years (Table 1). They had rather established disease with a median (interquartile range-IQR) time of disease duration of 57 (90) months. Only 18.5% of the patients started baricitinib as the first targeted therapy after failure of csDMARDs, while most of the patients received it after multiple ts/bDMARDs failures, thus baricitinib was the 3rd line of b/tsDMARDs. Almost half of them (51.1%) received baricitinib as monotherapy and continued as monotherapy throughout follow-up period. Apart from significant arthritis’ burden, 25% of the patients had a history of extra-articular manifestations with lung involvement being the most prevalent (6.7%).

**Table 1:** Baseline characteristics

Gender (females)	126 (93.3%)
Age Mean (SD)	56.1 (10.1)
Ever smoker^1^ (%)	42 (35.6%)
Education^2^	
Primary	26 (29.2%)
Secondary	34 (38.2%)
Tertiary	29 (32.6%)
Disease related characteristics	
Disease duration (months), median (min, max; IQR)	57 (1, 572; 90)
Baricitinib as line of treatment, median (min, max, IQR)	3 (1,8;2)
Baricitinib as 1st line of treatment	25 (18.5%)
RF positive^3^ (%)	30 (25.4%)
Anti CCP positive^4^ (%)	28 (25.0%)
Other rheumatic diseases	
Osteoarthritis any (%)	44 (32.6%)
Osteoporosis (%)	22 (16.3%)
Fibromyalgia(%)	41 (30.4%)
Treatments	
Monotherapy (%)	69 (51.1%)
Concomitant MTX (%)	38 (28.1%)
Concomitant LEF (%)	24 (17.8%)
Concomitant steroids (%)	38 (28.1%)
Extra-articular manifestations	
Any extra-articular (%)	34 (25.2%)
Pulmonary involvement (%)	9 (6.7%)
Rheumatic nodules (%)	7 (5.2%)
Secondary Sjogren’s (%)	3 (2.2%)
Pericarditis (%)	1 (0.7%)
Peripheral neuropathy (%)	1 (0.7%)
Cervical myelopathy (%)	1 (0.7%)
Chronic comorbidities	
Dyslipidemia (%)	46 (34.1%)
Hypertension (%)	42 (31.1%)
Obesity (%)	40 (29.6%)
Hypothyroidism (%)	36 (26.7%)
Depression (%)	18 (13.3%)
Type-II diabetes (%)	16 (11.9%)
Latent TB (%)	13 (9.6%)
GERD (%)	11 (8.1%)
NAFLD (%)	7 (5.2%)
Asthma (%)	6 (4.4%)

^1^17 missing values, ^2^46 missing/not reported, ^3^17 missing values, ^4^23 missing values

*TB* tuberculosis, *GERG* gastroesophageal reflux disease, *NAFLD* non-alcoholic fatty liver disease, *RF* rheumatoid factor, *anti-CCP* anti-citrullinated protein antibodies, *MTX* methotrexate, *LEF* leflunomide, *HCQ* hydroxychloroquine

Among comorbidities, metabolic diseases were common since 34.1% of the patients were treated for dylipidemia and 29.6% had obesity, while 31.1% received treatment for hypertension (Table 1). Almost 1/3 were active/ever smokers and 26.7% had hypothyroidism. Interestingly 13.3% of the patients received treatment for depression, almost 1/3 had fibromyalgia and 1/3 osteoarthritis.

At baseline, patients had active disease while all were on treatment with csDMSRDs or ts/bDMARDS. The mean DAS28 ESR was 4.91 (1.03) and 37.4% of them were on high DAS28 level (Table 2). Comparably to DAS28 all patients had active disease based on SDAI/CDAI [median 25.6 (17.8) & 25.0 (16.8) respectively] (Table 2). Patients scored high their functional disability [median mHAQ of 0.9 (0.9)] and the global status of their disease [median VAS general 60 (30)].

**Table 2:** Disease activity at baseline and evolution during at 6 and 12 months for patients under treatment at each time-point^1^

	Baseline (n=135)	6 months (n=111)	12 months (n=93)
DAS28 ESR [mean (SD)] Δ from baseline (95% CI)	4.83 (1.03) –	4.00 (1.28)* −0.76 (− 1.01 to −0.51)	3.62 (1.30)* −1.14 (− 1.43 to −0.85)
SDAI [median, min, max; IQR] Δ from baseline (95% CI)	25.6 (9.1, 75.0; 17.8)-	15.3 (2.7,48.3;15.2)* −9.00 (− 12.2 to −5.77)	14.2 (0.7, 31.2; 14.6)* −13.4 (− 17.2 to −9.49)
CDAI baseline [median, min, max; IQR] Δ from baseline (95% CI)	25.0 (7.0, 52.0; 16.8) –	15.0 (0.5,51.5;11.9)* −9.2 (− 11.7 to −6.7)	11.8 (0.5,32.0; 15.3)* −12.1 (− 15.0 to −9.1)
MDA (DAS28ESR)	62 (62.6%)	36 (46.2%)	26 (44.1%)
HDA (DAS28ESR)	37 (37.4%)	14 (17.9%)	7 (11.9%)
Swollen joint count (0–28) [median, min, max; IQR] Δ from baseline (95% CI)	7 (0, 19; 7) –	3 (0, 28; 4)* −2.9 (− 3.8 to −1.9)	2 (0, 13; 4)* −4.0 (− 5.2 to −2.9)
Tender joint count (0–28) [median, min, max; IQR] Δ from baseline (95% CI)	7 (0, 20; 6) –	2 (0, 20; 5)* −4.2 (− 5.3 to −3.0)	2 (0, 17; 4)* −4.6 (− 5.9 to −3.3)
mHAQ [median, min, max; IQR] Δ from baseline (95% CI)	0.90 [0.00, 1.80; 0.90] –	0.65 (0.00, 2.20; 0.75) −0.08 (− 0.19 to 0.03)	0.90 (0.00; 3.00; 1.30) 0.05 (− 0.10 to 0.18)
VAS general patient [median, min, max; IQR] Δ from baseline (95% CI)	60 [0, 100; 30] –	50 (0,95;35)* −9.9 (− 16.0 to −3.8)	52.5 (0,100;61)* −9.1 (− 16.2 to −1.9)
ESR (mm/h) [median, min, max; IQR] Δ from baseline (95% CI)	17.0 (0.0, 77.0; 18.0) –	18.0 (2.0, 65.0; 18.0) 0.56 (− 2.67 to 3.80)	15.0 (2.0, 74.0; 19.0) 0.83 (3.10 to 4.76)
CRP (mg/dL) [median, min, max; IQR] Δ from baseline (95% CI)	0.3 (0.1; 34.7; 0.7) –	0.3 (0.1; 16.0; 0.7) −0.13 (− 0.94 to 0.67)	0.2 (0.1,9.6; 0.4) −0.31 (− 1.29 to 0.66)

^*^Statistically significant differences compared to baseline (p<0.05) (linear mixed models)

^1^The following are the number of patients with available data at the respective time-points (baseline, 6 and 12 months respectively) for each-one of the parameters:

DAS28-ESR: n= 99, 88, 59

SDAI: n=91, 56, 34

CDAI: n=112, 70, 46

Swollen joint count (0–28): n=134, 77, 52

Tender joint count (0–28): n=134, 78, 53

mHAQ: n=111, 62, 3

VAS general patient: n=127, 74, 50

ESR: n=127, 70, 44

CRP: n=108, 66, 40

*DAS28* disease activity index of 28 joints count, *SDAI* simplified disease activity index, *CDAI* clinical disease activity index, *mHAQ* modified health assessment questionnaire, *VAS* visual analogue scale, *ESR* erythrocyte sedimentation rate, *cRP* c-reactive protein, *Δ from baseline* mean difference compared to baseline value

### Baricitinib retention and predictors thereof

**Fig. 1:**
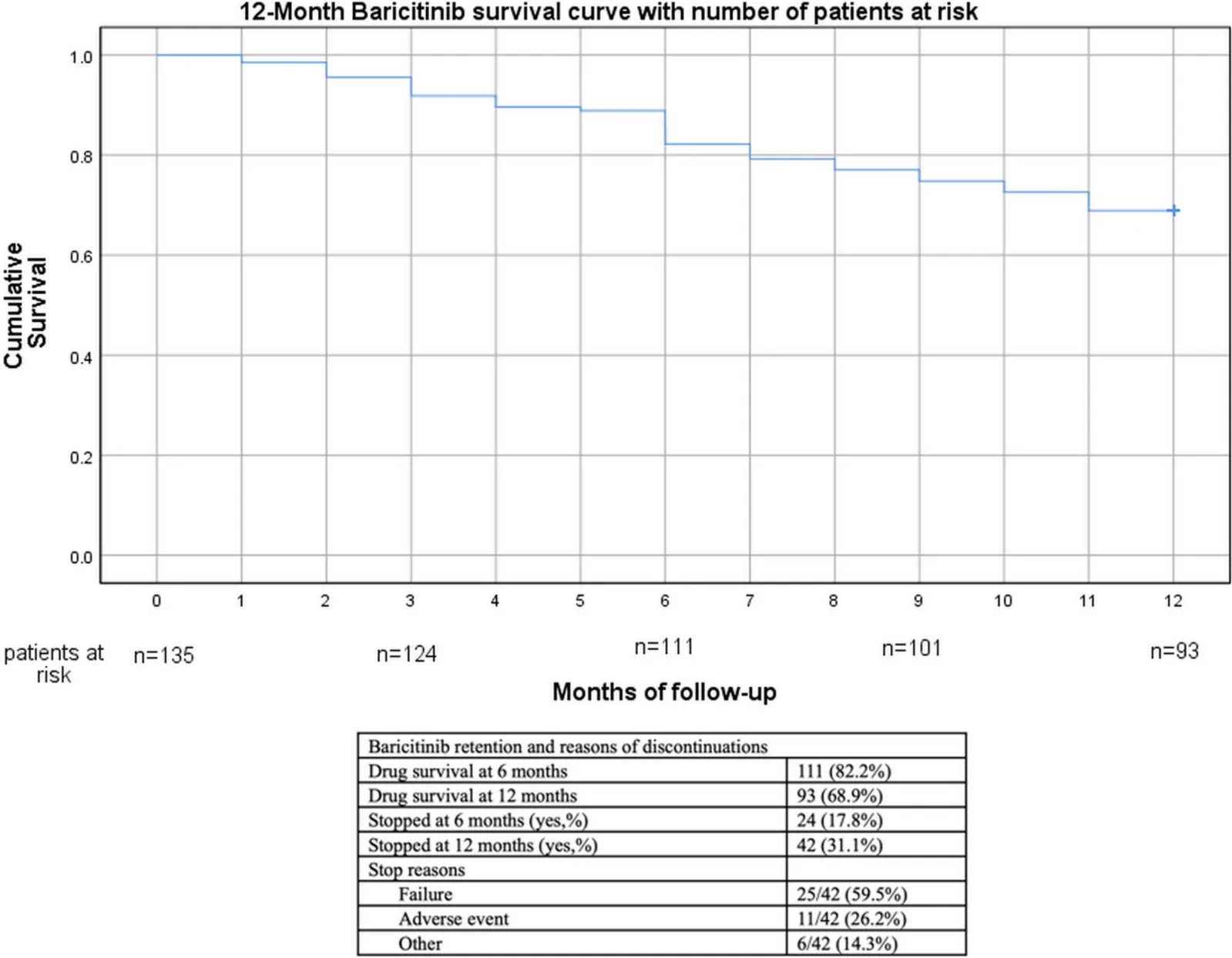
Baricitinib drug retention during 12 months of follow-up and reasons of discontinuation

We assessed baricitinib retention and it was shown that 93/135 patients (68.9%) remaind on baricitinib at 12 months. Almost half or the discontinuations (n=24/42) were recorded the first 6 months of treatment (17.8%) (Fig. 1). The main reason for discontinuation was failure (25/42, 59.5%), while 11/42 (26.2%) of the discontinuations were due to side effects and 6/42 (14.3%) due to other reasons.

**Fig. 2:**
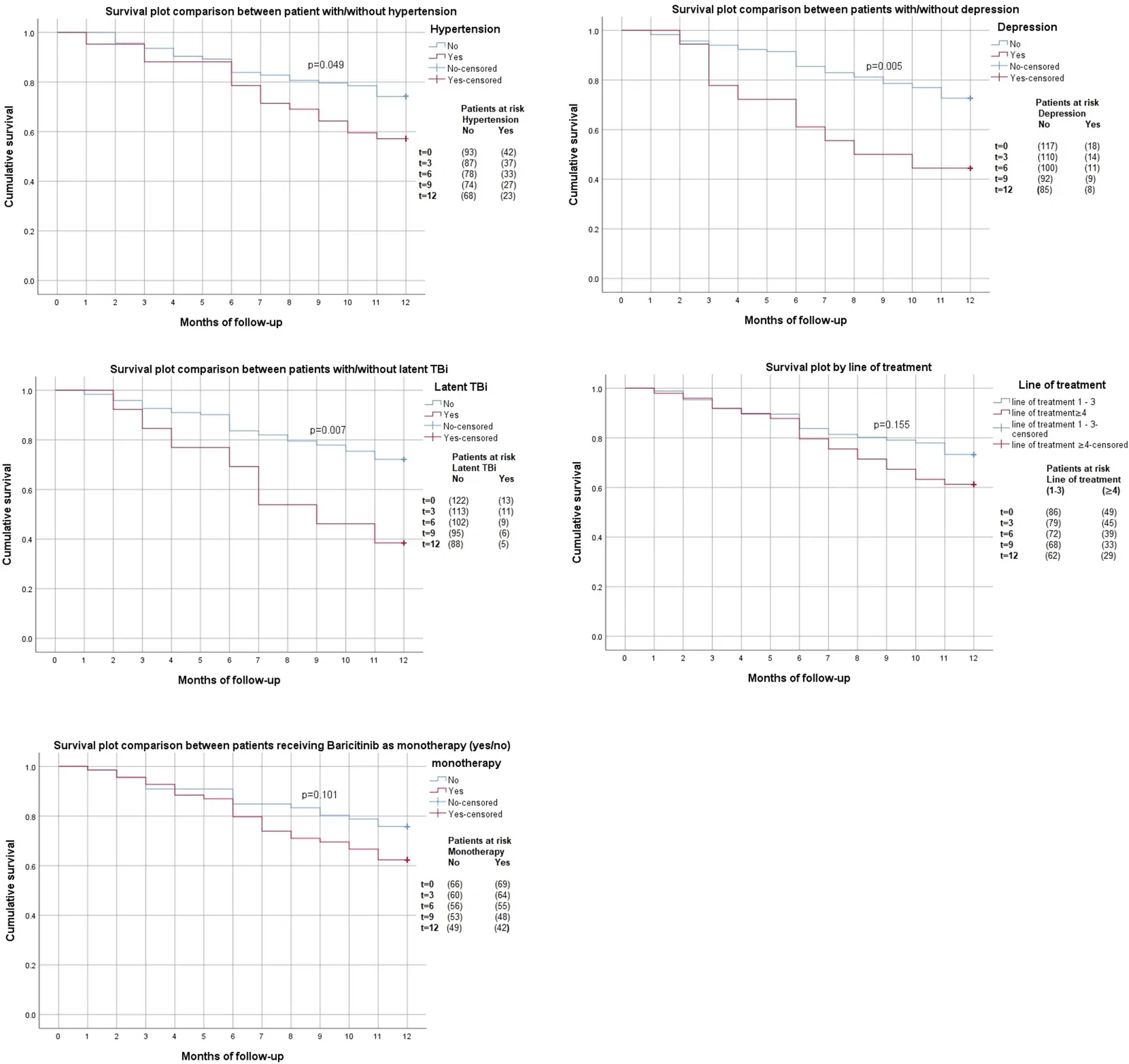
Baricitinib drug retention during 12 months of follow-up according to certain comorbidities and RA-related characteristics

We next assessed for disease-related factors and comorbidities to be associted with treatment retention. Initially we analysed all patients in the cohort. Univariate (Kaplan–Meier) analysis showed that while no disease-related factor was associtaed with baricitinib persistence at 12 moths, the presence of depression (p=0.005), LTBi (p=0.007) hypertension (p=0.049), were associated to a shorter persistence of the drug (Table 3 and Fig. 2). Since it has been shown that early (at first 6 months) disease control is associated to future response/drug survival, we repeated the analysis accounting for disease activity at 6 months and including only those patients who stopped beyond the first 6 months of therapy. Univariate (Kaplan–Meier) analysis revealed that starting baricitinib with high disease activity (HDA) (p=0.041) and not achieving remission or low disease activity (LDA) at 6 months (p=0.014) were the RA-related characteristic to be associated with baricitinib discontinuaton at 12 months (Supplementary Table 1). Interestingly, among the comorbid diseases it was shown that LTBi (p=0.008) and hypertension (p=0.03) were associated to shorter baricitinib retention (Supplementary Table 1).

**Table 3:** Kaplan Meier univariate survival analysis for drug retention at 12 months for the total group

Line of treatment	1–3	≥4	p-value
Cumulative proportion (SE)	0.733 (0.048)	0.612 (0.070)	0.155
Survival time Mean (95% CI)	10.4 (9.7–11.1)	9.8 (8.9–10.7)	
Monotherapy	No	Yes	p-value
Cumulative proportion (SE)	0.758 (0.044)	0.623 (0.058)	0.101
Survival time Mean (95% CI)	10.5 (9.8–11.3)	9.9 (9.1–10.6)	
HDA at baseline	No	Yes	p-value
Cumulative proportion (SE)	0.746 (0.053)	0.593 (0.067)	0.070
Survival time Mean (95% CI)	10.5 (9.8–11.2)	9.6 (8.6–10.5)	
Hypertension	No	Yes	p-value
Cumulative proportion (SE)	0.742 (0.045)	0.571 (0.076)	0.049
Survival time Mean (95% CI)	10.5 (9.9–11.1)	9.5 (8.5–10.6)	
Depression	No	Yes	p-value
Cumulative proportion (SE)	0.726 (0.041)	0.444 (0.117)	0.005
Survival time Mean (95% CI)	10.5 (10.0–11.0)	8.2 (6.4–10.0)	
Latent TBi	No	Yes	p-value
Cumulative proportion (SE)	0.721 (0.041)	0.385 (0.135)	0.007
Survival time Mean (95% CI)	10.4 (9.8–10.9)	8.4 (6.4–10.4)	

*SE* Standard error, *CI* confidence intervals, *LDA* Low disease activity (DAS28), *HDA* high disease activity, *TBi* tuberculosis infection

We then performed multivariate backward Cox regression analysis, for factors predicting baricitinib discontinuation at 12 months. As above, initial analysis focused on the total group, in which it was shown that the presence of depression [hazard ratio (HR) (95% CI) 2.715 (1.313–5.614), p=0.007] and LTBi [HR 2.519 (95% CI) (1.154–5.499), p=0.020] were significant predictors for stopping baricitiniob at 12 months (Table 4). We further assessed for predictors in patients stopping baricitinib after the 6th month of therapy. It was shown again as in the univariate analyisis, that disease activity levels at 6 months [HR (95% CI) 1.434 (1.049–1.961), p=0.024], together with hypertension [HR (95% CI) 2.477 (1.137–5.395), p=0.022] and LTBi [HR (95%CI) 3.761 (1.198–11.25), p=0.023] were all significant contributors to baricitinib discontinuation (Supplementary Table 2).

**Table 4:** Backward Cox regression model predicting the hazard ratios of stopping baricitinib at 12 months for all patients of the cohort

Predictor	Hazard ratio	95% CI	p-value
Hypertension (yes)	2.186	0.975–4.898	0.058
Depression (yes)	2.715	1.313–5.614	0.007
Latent TBi (yes)	2.519	1.154–5.499	0.020

Chi-square 18.834 on d.f. 3; p<0.0001, n=135, 39 events, 14 cases with missing values, n=121 cases included in the model, events-per-variable = 13.0. No case exerted disproportionate influence on model coefficients (DFBeta diagnostics)

*TBi* tuberculosis infection

### Disease activity evolution and safety

Concerning disease activity, baricitinib improved DAS28 ESR at both 6 and 12 months statistically significantly (p<0.05) and close to clinically significant level for the total group at 12 months (difference to baseline 1.14) (Table 2). Comparable to DAS28, disease activity was significantly improved as assesed by both SDAI and CDAI (p<0.05 for both indexes at 6 and 12 months) (Table 2). Interestingly 35.9% (28/88) 44.1% (26/59) of the patients under follow-up attained remission/LDA at 6 and 12 months respctively.

Patients were followed for a total of 114.67 person-years. During this period a total of 62 adverse events (AEs) including mild (n=27) (Supplementary Table 3), moderate (n=32) and serious (n=4) were recorded in 45 patients. The incidence rate (IR) of the 32 moderate AE was 279.1 (95% CI 191.0 − 394.0) per 1000 person-years of observation. 16 out of 32 moderate AEs were infections [IR: 139.5 (95%CI 79.8–226.6 per 1000 person-years], and 1 was a moderate cardiovascular (CVD) (arrythmia) [IR 8.7 (95%CI 0.22–48.6) per 1000 person-years]. Only 4 of the AEs were serious [IR: 34.9 (95% CI 9.5–89.3) per 1000 person-years], with 2 serious infections (pyelonephritis, upper respiratory tract infection) [17.4 (95%CI 2.11 to 63.0) per 1000 person-years] and 1 serious CVD event (hemorrhagic stroke) [IR 8.7 (95%CI 0.22–48.6) per 1000 person-years]. The 4th serious AE was “radiculopathy/herniated disc” for which **“**spinal fusion” surgery was done.

## Discussion

In this real-world, multicenter cohort study of 135 patients with RA treated with baricitinib, we found drug retention rates of 82% at 6 months and 69% at 12 months, in a “heavily” pretreated population (median treatment line of targeted therapies n=3). Interestingly disease-related and comorbidities differentially affected drug survival according to time of discontinuation: depression and LTBi were significant contributors to baricitinib retention during the total 12 months of follow-up, while disease activity levels at 6 months, the presence of hypertension and LTBi affected later than 6 months discontinuations. Baricitinib demonstrated clinically relevant improvement in disease activity.

In our cohort we observed a retention rate of 69% at 12 months, while half of the discontinuations were recorded during the first 6 months of treatment (Fig. 1). The main reason of discontinuations was ineffectiveness (59.5% of discontinuations) while 26.2% were due to safety reasons. This retention rate is comparable to what it has been reported in the literature, which ranges between 66.5% and 70% at 12 months, while for 24 months it has been reported up-to 61.8% [13–15, 17]. Thus, a rather constant retention rate has been shown in the above reported international registries despite differences in patients’ and disease’s background.

Retention rate in the total cohort was found to be independent of several RA related factors, like seropositivity, disease duration or co-administered DMARDs (Table 3 and Fig. 2). The rather low number of patients included in our analysis could be a reason of absence of significant association between RA-related factors and drug survival in the total cohort, although and comparable to our findings, no strong association between disease-related characteristics and baricitinib survival were reported in an analysis by the BSRBR-RA registry [13]. Comorbidities have been shown to affect rheumatoid arthritis outcome and especially drug retention [18–21]. Concerning JAK inhibitors, data from real-life cohorts have focused mostly in cardiovascular comorbidities and their contribution in safety, while no solid data about general comorbidities and their effect in drug retention rates are available [22]. Herein, the univariate analysis showed that at baseline presence of depression, LTBi and hypertension were significantly associated to baricitinib survival (Table 3 and Fig. 2). LTBi has been shown to be associated with shorter bDMARDs drug survival in endemic areas like Taiwan even recently [23]. There was no case of TB reactivation and all LTBi patients were treated with prophylactic therapy. We consider that these patients represent a group of patients with a higher disease- and comorbidities-related burden. Interestingly, in multivariate analysis of baricitinib persistence in the total cohort, only depression was found as a significant contributor (HR 3.225 (1.380–7.537), p=0.007) (Table 4). Specifically for depression, it has been associated to bDMARDs discontinuation in registry studies [18, 24]. In a recent review paper summarizing published data in the field of D2T RA, depression was reported to be associated with D2T in 9/21 studies (43%) [25]. Since patients of our cohort received baricitinib as the 3rd line targeted therapy, our data corroborate the above-mentioned data for the contribution of depression in a lower retention rate.

Given that early responses have been shown to be associated to drug persistence, we performed the above analysis accounting also for clinical responses at 6 moths and thus censoring the analysis for patient who remained >6 months on baricitinib (Supplementary Table 1 & 2) [26]. Lower disease activity both at baseline and at 6 months were associated to baricitinib survival at 12 months (Supplementary Table 1). Concerning comorbidities we found that hypertension (p=0.030) and LTBi (p=0.008) were associated to baricitinib persistence for 12 months, while depression was not (Supplementary Table 1). This could be due to the smaller number of patients available for this group as compared to the total cohort, or to a “true” association of depression with earlier (during the first 6 months) discontinuation risk. Interestingly, multivariate analysis confirmed that together with comorbidities (hypertension and LTBi) higher DAS28 at 6 months was a significant contributor to baricitinib discontinuation (Supplementary Table 2). It has been shown that achieving remission/LDA and generally effective disease control at 3 or 6 months of bDMARDs initiation is associated to drug persistence [26]. Furthermore, in a recent analysis for D2T RA from Greece, it has been shown that in patients starting targeted therapies achievement of clinically significant improvement at 6 months (δDAS28>1.2) was an independent factor of not evolving to D2T [27]. On could claim that depression is a major contributor to early (first 6 months) discontinuations of baricitinib, while later disease control and other comorbidities have a role. Nevertheless, whether a true differential contribution of comorbidities in reference to time of discontinuation of baricitinib should be assessed in larger studies.

The strength of our analysis is that this is a multicenter study of patients representative of the RA population treated in clinical practice, concerning the presence of comorbidities, extra-articular disease and a rather high burden of prior csDMARDs and bDMARDs exposure. Moreover, this is the first real-life analysis of baricitinib retention with a special focus in comorbidities, since although there are a lot of relevant studies for RA patients treated with bDMARDs, literature for baricitinib is limited. Nevertheless, the study has several limitations. First, the absence of a control group and the observational design cannot establish causality between comorbidities, disease activity and discontinuation for baricitinib. Second, the wide confidence intervals around several hazard ratios, particularly for hypertension, depression and latent tuberculosis, reflect the relatively small number of discontinuation events in these subgroups and limit the precision of our estimates; larger or pooled cohorts are needed to confirm whether these associations are fully explained by disease activity or represent independent effects not captured by our covariates.

In this real-world, “heavily” pretreated RA cohort, baricitinib produced clinically relevant and sustained reductions in disease activity, with roughly two-thirds of patients remaining on treatment at 12 months. The differential contribution of depression in baricitinib survival, significant only for discontinuations within the first 6 months of treatment, although interesting cannot be explained based on our cohort’s data. Early clinical response and selected comorbidities but not depression were the strongest independent drivers of drug survival beyond the 6th month of treatment. Should these findings be confirmed in other cohorts a special attention to disease control and specific comorbidities could improve baricitinib use in clinical practice.

## Supplementary Information

Below is the link to the electronic supplementary material.

Supplementary file1 (DOCX 22 KB)
