# Supplementary material for: Baricitinib retention and factors associated thereof in clinical practice: a prospective, multicenter 12-month study in rheumatoid arthritis patients
Source: Rheumatol Int. 2026 Oct 5;46(10):276. doi: 10.1007/s00296-026-06313-y (PMC13638749; doi:10.1007/s00296-026-06313-y)
Supplement: Supplementary file 1 [file 296_2026_6313_MOESM1_ESM.docx]

**SUPPLEMENTARY**

| **Supplementary Table 1**. Kaplan Meier univariate survival analysis for drug retention at 12 months for patients continuing baricitinib for>6 months. | | | |
| --- | --- | --- | --- |
| **Line of treatment** | **1-3** | **≥4** | **p-value** |
| Cumulative proportion (SE) | 0.875 (0.039) | 0.769 (0.067) | 0.130 |
| Survival time  Mean (95% CI) | 11.7 (11.4 – 11.9) | 11.3 (10.8 – 11.7) |  |
| **Monotherapy** | **No** | **Yes** | **p-value** |
| Cumulative proportion (SE) | 0.893 (0.041) | 0.782 (0.056) | 0.104 |
| Survival time  Mean (95% CI) | 11.8 (11.5 – 11.9) | 11.3 (10.9 – 11.7) |  |
| **LDA/remission at 6 months** | **No** | **Yes** | **p-value** |
| Cumulative proportion (SE) | 0.740 (0.062) | 0.964 (0.035) | 0.014 |
| Survival time  Mean (95% CI) | 11.2 (10.7 – 11.6) | 11.9 (11.8 – 12.0) |  |
| **HDA at baseline** | **No** | **Yes** | **p-value** |
| Cumulative proportion (SE) | 0.893 (0.041) | 0.744 (0.067) | 0.041 |
| Survival time  Mean (95% CI) | 11.8 (11.6-12.0) | 11.1 (10.6 – 11.6) |  |
| **Hypertension** | **No** | **Yes** | **p-value** |
| Cumulative proportion (SE) | 0.885 (0.036) | 0.727 (0.078) | 0.030 |
| Survival time  Mean (95% CI) | 11.7 (11.5 – 11.9) | 11.1 (10.5 – 11.7) |  |
| **Depression** | **No** | **Yes** | **p-value** |
| Cumulative proportion (SE) | 0.850 (0.036) | 0.727 (0.134) | 0.245 |
| Survival time  Mean (95% CI) | 11.6 (11.4 – 11.8) | 11.0 (10.0 – 12.0) |  |
| **Latent TB** | **No** | **Yes** | **p-value** |
| Cumulative proportion (SE) | 0.863 (0.034) | 0.556 (0.166) | 0.008 |
| Survival time  Mean (95% CI) | 11.6 (11.4 – 11.8) | 10.4 (9.1 – 11.8) |  |
| SE: Standard error; CI: confidence intervals; LDA: Low disease activity (DAS28); HDA: high disease activity; TB: tuberculosis | | | |

| **Supplementary Table 2**. Backward Cox regression model predicting the hazard ratios of stopping baricitinib at 12 months for patients continuing baricitinib for>6 months | | | |
| --- | --- | --- | --- |
| **Predictor** | Hazard ratio | 95% CI | p-value |
| DAS28 ESR at 6 months | 1.434 | 1.049 – 1.961 | 0.024 |
| Hypertension (yes) | 2.477 | 1.137 – 5.395 | 0.022 |
| Latent TBi (yes) | 3.761 | 1.198 – 11.25 | 0.023 |
| Chi-square 20.370 on d.f. 3; p<0.0001; n=111, 27 events, 33 cases with missing values, n=78 cases included in the final model  TBi: tuberculosis infection | | | |

| **Supplementary Table 3.** Description of mild adverse events | | |
| --- | --- | --- |
| **System** | **Description** | **number** |
| Infections and infestations | 7 Upper respiratory track  1 Urinary tract infection  1 Stomatitis  1 Gastroenteritis  1 Mucosal infection | 11 |
| Blood and lymphatic system disorders | 1 Generalized lymphadenopathy, resolved with no treatment.  1 Thrombophilia  1 Leukopenia possibly due to leflunomide  1 anemia | 4 |
| Skin and subcutaneous tissue disorders | Skin rash | 2 |
| Musculoskeletal and connective tissue disorders | Osteoporosis  Osteoarthritis  Carpal tunnel syndrome | 4 |
| Gastrointestinal disorders | Diarrhea, resolved | 2 |
| Renal and urinary disorders | Chronic kidney disease  Hematuria | 2 |
| Psychiatric disorders | Emotional disturbance/ depression | 1 |
